# Prevalence and Risk Factors of Musculoskeletal Pain Among Kuwaiti Pilgrims During Hajj 2024

**DOI:** 10.3390/ijerph22101585

**Published:** 2025-10-18

**Authors:** Tahra Aleid, Nowall Al-Sayegh, Sultan E. Alsalahi, Abdulaziz Alhenaidi

**Affiliations:** 1 Department of Physical Therapy, Alrazi National Orthopaedic Hospital, Ministry of Health, Sulaibkhat, Jamal Abdulnasser Street, Safat 13001, Kuwait; taleid@moh.gov.kw; 2 Department of Physical Therapy, Allied Health Sciences College, Kuwait University, Safat 13001, Kuwait; nowall.alsayegh@ku.edu.kw; 3 Directorate of Planning and Monitoring, Department of Planning and Monitoring, Ministry of Health, Safat 13001, Kuwait; aalhenaidi@moh.gov.kw; 4 School of Health and Wellbeing, University of Glasgow, GPPC, 124 Observatory Road, Glasgow G12 8UZ, UK

**Keywords:** musculoskeletal pain, Hajj, Kuwait, pilgrims, risk factors, physical activity, sleep, smoking

## Abstract

Background: Musculoskeletal pain (MSP) is one of the leading causes of disability worldwide and is frequently reported during the Muslim Hajj Pilgrimage; however, its prevalence and associated risk factors among Kuwaiti pilgrims have not been studied thus far. Methods: This is a retrospective cross-sectional study of Kuwaiti pilgrims conducted during the year 2024. Pilgrims were contacted by phone before and after Hajj to answer a survey regarding MSP during their pilgrimage. Risk ratios were computed using binomial generalised linear models with a log link. Results: A total of 557 participants (Mean BMI 28.0 ± 8.0 kg/m^2^), comprising 340 women (61%) and 217 men (39%), participated in the study. Most were between 33 and 45 years of age (*n* = 173, 31%), with 24% of the sample (*n* = 136) reporting MSP. Our regression analysis revealed that female gender (aRR 1.49, 95% CI 1.08–2.06), short sleep duration (<6 h; aRR 1.37, 95% CI 1.02–1.84), and smoking (aRR 0.66, 95% CI 0.46–0.95) were significantly associated with MSP, while participants who did not report hypertension were also less likely to report MSP (aRR 0.64, 95% CI 0.46–0.89). Conclusions: This study, the first to focus on Kuwaiti pilgrims in this regard, showed that their reported prevalence of MSP during Hajj was lower than reported previously in studies of other nationalities. Several factors that increased the risk of MSP included smoking, hypertension, poor sleep, and female gender. The results of this study emphasise the necessity of both conducting a screening programme before Hajj and raising awareness of the factors that increase the prevalence of MSP, subsequently reducing the readiness of pilgrims.

## 1. Introduction

MSP is one of the leading causes of disability worldwide and is defined as discomfort in the anatomical structures that support movement [1,2,3]. Sudden physical exertions increase the chances of developing MSP, mostly due to inadequate rest, repetitive movements, and abnormal load distribution [4,5]. The Muslim Hajj Pilgrimage presents pilgrims with a physically demanding array of rituals that involve walking more than 10 kilometres each day for several days, within a short timeframe and in high temperatures. Understanding factors that contribute to MSP during physically demanding events is crucial for risk minimisation.

In order to mitigate the risk of MSP during events that place physical strain on participants, physical preparedness is key. This can involve gradually increasing the intensity of physical activity, engaging in a structured warm-up routine, and incorporating strength and mobility exercises [6,7]. As reported elsewhere, another factor that can help mitigate the risk of MSP during demanding physical activity is sleep duration, given the crucial role of sleep in bodily recovery [8,9]. Other risk factors that are implicated in increased MSP incidence are increased body fat percentage and hypertension [10,11,12]. Therefore, prioritising physical preparation and focusing on risk factors can mitigate MSP risks during physically demanding events such as Hajj.

Few studies have investigated the prevalence of MSP during Hajj, but all have reported it as a common concern among pilgrims [13,14,15,16]. Previous studies have reported a high prevalence of MSP in this context, ranging from 70 to 80% across multi-national cohorts [13,14,15,16]. It is well known that sudden increases in physical demand and biomechanical overload result in increases in MSP [4,17]; therefore, investigating the prevalence of Hajj-related MSP is vital. Alshehri, Alzaidi [14] reported over 80% prevalence, with the most affected regions being the ankle/foot, leg, lower back, and knee. Similarly, Alghamdi, Alghamdi [13] reported that 80% of participants experienced musculoskeletal injuries, while Marashi, Rusta [15] reviewed the medical records of over 100,000 Iranian pilgrims and found that the primary cause of visits to physicians was MSP. To our knowledge, no study has yet investigated the prevalence of MSP among Kuwait pilgrims.

Understanding pain patterns and risk factors is paramount for establishing national prevention programmes in order to educate pilgrims before Hajj and mitigate the risk of MSP. Moreover, this will help alleviate the stress on the medical teams available on site, allowing them to focus on more demanding conditions that may arise during Hajj. Factors such as age, gender, and pre-existing conditions have all been reported as risk factors of MSP during Hajj [13,14,16]. However, given the research gap regarding the prevalence and patterns of MSP in Kuwaiti pilgrims, we aimed to investigate this cohort in the 2024 Hajj. Unique factors among Kuwaiti pilgrims may introduce differential risk profiling compared to multi-national cohorts due to differences in baseline physical preparedness and health profiling. Indeed, Alghamdi, Alghamdi [13] found significant differences in pain reporting between pilgrims, with international pilgrims being more likely to report MSP during Hajj. Therefore, by examining a national cohort, our research can be used to develop targeted interventions for Kuwaiti pilgrims and enhance Hajj preparedness.

## 2. Methodology

This is a cross-sectional study of Kuwaiti pilgrims conducted in the year 2024. Pilgrims were contacted by phone; pre-Hajj data collection was conducted from March to May, and post-Hajj recall was conducted from July to November.

### 2.1. Participant Recruitment

The Ministry of Awqaf and Islamic Affairs was approached to compile a list of pilgrims who would be joining the 2024 pilgrimage. A group of volunteers assisted in contacting pilgrims before and after the commencement of the Hajj. Volunteers received standard training and instructions on how to approach and record survey responses. The first round of data collection took place before Hajj during April and May of 2024, and the second round commenced after Hajj during the period between July and August.

### 2.2. Survey Instrument

The survey consisted of three sections (Supplementary File S1): demographics, health information (including musculoskeletal information), and lifestyle information. The first section consisted of questions regarding participants’ demographics, including gender, age, marital status, education, and weight. The second section consisted of questions related to the participants’ current health, and if they suffered from any medical conditions, including female-specific conditions where appropriate. Participants were also asked about any musculoskeletal conditions or pain at any site in the body. The final section pertained to participants’ lifestyles, including questions on whether they exercised, smoked, or used a wheelchair for daily movement, in addition to their sleep duration. After Hajj, MSP was self-reported by asking participants whether they had experienced any during the pilgrimage, and the location of the pain, if present. No external devices or other instruments were used to report MSP. Participants were also asked if they had used a wheelchair during Hajj, whether they had any injuries, and if so, what medications they had used for their pain or injuries. Additionally, they were asked about their sleep duration after Hajj.

### 2.3. Sample Size Calculation

A total of 8000 Kuwaiti pilgrims registered to join the Hajj during the year 2024. Assuming a 5% margin of error and a 95% confidence interval, 367 participants were required. Participants were randomly selected based on convenience with no stratification beyond selection.

### 2.4. Ethical Considerations

This study protocol was reviewed and approved by the Ministry of Health of Kuwait Ethical Committee (2017/650). During the phone call, participants were first asked to provide verbal informed consent after being informed about the study and what they could expect to gain from participating.

### 2.5. Statistical Analysis

The data was transcribed into a digital format, exported to Excel for further analysis, and then cleaned. Participants with incomplete data were excluded from the analysis. Besides descriptive statistics, regression analysis was conducted using a binomial generalised linear model with a log link function to measure risk ratios (RRs): whether a specific exposure is a risk factor for a specific outcome [18,19]. RR results were grouped into one of three categories: ‘exposure does not affect the risk of outcome’ (R = 1), ‘exposure is associated with lower risk’ (R < 1), and ‘exposure is associated with higher risk’ (R > 1) [14]. Predictors were included in regression based on previous Hajj studies on MSP [13,14,16]. Crude risk ratios were obtained using univariate regression analysis, while adjusted risk ratios were derived from multiple regression analysis, adjusting for sex, age, hypertension, smoking, sleep, moderate exercise, and musculoskeletal condition. In addition, to assess whether the predictors of MSP were correlated with the condition or not, we tested each predictor for multicollinearity using variance inflation factor and tolerance statistics. A tolerance value of <0.2 or VIF > 5 was considered indicative of multicollinearity with MSP and therefore eliminated from regression analysis. All analyses were performed using the Statistical Package for the Social Sciences (SPSS IBM version 29.0). Results were considered statistically significant when *p* < 0.05.

## 3. Results

### 3.1. Demographics

**Figure 1:**
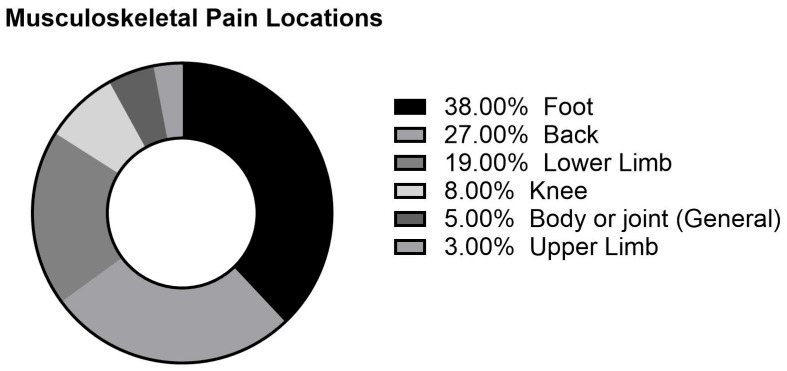
Locations of musculoskeletal pain reported by pilgrims during Hajj.

A total of 557 responses were included after removing missing and incomplete responses (Table 1). The sample comprised 340 women (61%) and 217 men (39%). Most participants were aged between 33 and 45 years (*n* = 173, 31%), with those aged under 33 comprising the next largest group (*n* = 153, 27%). The mean BMI of the sample was 28.0 ± 8.0 kg/m^2^. In total, 13% of participants reported having diabetes, 14% reported having hypertension, and 27% reported having a musculoskeletal condition. Additionally, 39% of the sample reported engaging in moderate physical activity, 30% reported sleeping less than 6 h, and 14% reported smoking. The post-Hajj survey revealed a 24% incidence of MSP, while 5% of the participants reported sustaining musculoskeletal injuries during Hajj. Furthermore, 19% of participants reported taking medication, 35% noted the need for assistance with movement during Hajj, and the percentage of those sleeping for less than 6 h rose to 80%, compared to 30% before Hajj commencement. Figure 1 demonstrates the distribution of reported MSP locations during Hajj. The most common location was the foot, which accounted for 38% of MSP, followed by the back (27%) and lower limbs (19%).

### 3.2. Correlation and Regression

Table 2 demonstrates the results of multivariable binomial regression with log link. Variables were tested for multicollinearity before regression analysis, with none meeting the threshold for elimination. Univariable analysis was used to examine the crude association (model 1) of all predictors of MSP. Women (crude RR 1.43, 95% CI 1.04–1.97; *p* = 0.030) and participants who slept for under 6 h (crude RR 1.45, 95% CI 1.08–1.94; *p* = 0.014) were at higher risk of MSP, while participants without hypertension (crude RR 0.65, 95% CI 0.46–0.93; *p* = 0.017) or existing musculoskeletal conditions (crude RR 0.67, 95% CI 0.50–0.91; *p* = 0.009 were at a lower risk of MSP.

This higher risk of MSP for women (aRR 1.49, 95% CI 1.08–2.06; *p* = 0.015) and individuals sleeping less than 6 h (aRR 1.37, 95% CI 1.02–1.84; *p* = 0.038) was also demonstrated by multivariable binomial regression; in this analysis, participants without hypertension (aRR 0.64, 95% CI 0.46–0.89; *p* = 0.009) and non-smokers (aRR 0.66, 95% CI 0.46–0.95; *p* = 0.027) were also associated with a lower risk of MSP. No other variables were significant predictors of MSP (*p* > 0.05).

## 4. Discussion

In this study, we investigated the prevalence of MSP and related risk factors among Kuwaiti adult pilgrims during the Hajj. The main findings of this study are two-fold. First, 24% of participants reported experiencing MSP during Hajj. Secondly, an increased risk of MSP was significantly associated with the modifiable factors of smoking, hypertension, and sleep, as well as the non-modifiable factor of gender.

### 4.1. Prevalence of MSP

This study contrasts previously published results regarding MSP prevalence during Hajj [13,14,16]; the 24% rate of MSP that we report is lower than that reported in previous studies that focus on the same region. Alshehri, Alzaidi [14], Alghamdi, Alghamdi [13], and Alsobhi and Aldhabi [16] all reported that 78–80% of participants complained of MSP during Hajj. These differences may be attributed to variations in the methodologies used to report pain. Indeed, our study involved the retrospective recall of pain that occurred during Hajj, which might have led to under-reporting of the prevalence of MSP, while the studies mentioned previously involved on-site data collection and may have over-reported the incidence of acute MSP [20,21,22]. However, a study of the 2010 Hajj reported an extremely low pain rate (7%) [15], while a further study of a mass religious gathering event in Iraq that involved walking for long distances reported a joint pain rate of 28% [23]. Studies that have investigated the prevalence of MSP through community-based surveys have reported consistently lower prevalence rates (15–21% MSP) in Sweden, Brazil, and Japan [24,25,26]. Moreover, in a systematic review and meta-analysis, the prevalence of MSP in low- and middle-income countries was around 33% in general adults and over 56% in older adults [27]. This consistency reinforces the credibility of the methods used in our study. Other contributors to these discrepancies might be related to cultural differences in reporting pain [28,29]. Indeed, our study only included Kuwaiti adults, while other studies have included MSP reports from multi-national samples during Hajj [13,14,16]. Additionally, nationals from high-income countries such as Kuwait may opt for travel arrangements that reduce the amount of walking required and ensure better transport within the event, leading to lower rates of MSP being reported than in other multi-national cohorts. However, daily steps or travel data were not available in this study, preventing further comparisons from being made. Future studies may quantify the impact of travel arrangements on pilgrim health during Hajj in a national sample.

### 4.2. Risk Factors

This study has revealed several factors that increase the incidence of MSP during Hajj. Female gender was linked to a higher risk of MSP, in agreement with previous studies that showed a higher prevalence of MSP in women than in men [14,24,25,26]. This may be attributed to gender differences and variations in pain perception and sensitivity [30,31,32]. Notably, hormonal influences and gene variations have been implicated in sex-oriented pain perception and sensitivity [30]. Other factors that may increase the risk of developing MSP during Hajj include smoking, short sleep duration (<6 h), and hypertension. Earlier reports have confirmed the prevalence of pain among people with hypertension [33,34]; therefore, pain management strategies should be used to help avoid MSP during Hajj, especially among vulnerable populations [34]. It is likely that poor vascular health and baroreceptor dysfunction following heightened blood pressure contribute to pain sensitivity, while altered microvascular function and structure can sensitise pathways that are linked to pain [35,36]. Furthermore, poor sleep has also been linked to pain in the literature [37,38,39,40]; Goossens, Van Stallen [39] indicated that poor sleep can have a direct effect on pain intensity the following day in patients with chronic MSP. This link may be attributed to sleep deprivation disrupting pain receptors or the pain inhibitory process [41,42,43]. With regard to our finding that smokers were more likely to report MSP than non-smokers, previous studies have also identified increases in MSP in smokers compared to non-smokers [44,45,46]. Potential mechanisms may include the negative effects of smoking on bone and muscle healing processes [47] and the related increased risk of pain in the limbs and joints (OR:1.16–1.26, respectively) [48].

### 4.3. Policy Implications

Although these associations have potential implications for prevention strategies, our study design limits causal inference. However, several interventions can help manage MSP and alleviate stress on medical professionals during Hajj using pre-screening and physiotherapy. Firstly, musculoskeletal screening is crucial before participation in activities that involve physical demands, such as Hajj [49]. It is advisable to inform participants of their current conditions and, if necessary, to address them by providing workshops led by professionals (such as physiotherapists) to promote awareness, or by implementing a screening clinic and promoting physical activity before Hajj [50]. Integrating physiotherapy services and providing access to physiotherapists is advisable during the Hajj process; this can offer a route to relieve injuries and pain [51].

### 4.4. Study Limitations and Future Directions

While our results provide valuable insights regarding Hajj and MSP, the reliance on self-reported data may have caused recall bias and led to underestimating the prevalence of MSP. Future studies should investigate the effect of conducting a pre-Hajj programme on MSP during Hajj by using other objective means of pain reporting, such as the Visual Analogue Scale or Oswestry Disability Index [52,53,54]. Furthermore, given the study’s cross-sectional design, the causality between Hajj and MSP cannot be confirmed. Future studies should focus on investigating the impact of increasing physical activity or implementing an intervention, such as structured exercises, prior to performing Hajj. This can aid in understanding how increased activity may reduce the incidence of MSP during demanding activities.

## 5. Conclusions

This study demonstrated the prevalence of MSP in Kuwaiti pilgrims during Hajj and revealed several factors that increase risk, such as smoking, hypertension, poor sleep, and the greater susceptibility of women to MSP compared to men. The results of this study emphasise the necessity of both implementing a screening programme before Hajj and raising awareness of the factors that increase the prevalence of MSP and reduce the readiness of pilgrims.

## Figures and Tables

**Table 1:** Characteristics of study participants (*n* = 557). Values are represented as Frequencies (*n*) and percentages (%). Hrs = hours.

Variable		*n* (%)
Age group	<33 years	153 (27%)
	33–45 years	173 (31%)
	46–60 years	140 (25%)
	60+ years	91 (16%)
Gender	Female	340 (61%)
	Male	217 (39%)
Diabetes	No	479 (86%)
	Yes	78 (14%)
Hypertension	No	486 (87%)
	Yes	71 (13%)
Any musculoskeletal conditions?	No	404 (73%)
	Yes	153 (27%)
Any medical conditions?	No	279 (50%)
	Yes	278 (50%)
Wheelchair usage?	No	503 (90%)
	Yes	54 (10%)
Moderate exercise	No	339 (61%)
	Yes	218 (39%)
Do you smoke?	No	477 (83%)
	Yes	80 (14%)
Post-Hajj		
Sleeping duration	<6 hrs	167 (30%)
	>6 hrs	390 (68%)
Pain	No	421 (76%)
	Yes	136 (24%)
Injury	No	528 (95%)
	Yes	29 (5%)
Sleeping duration	<6 hrs	444 (80%)
	>6 hrs	113 (20%)
Medications	No	449 (81%)
	Yes	108 (19%)

**Table 2:** Crude and adjusted risk ratios (RRs) with 95% confidence intervals and *p*-values for predictors of musculoskeletal pain prevalence. To obtain risk ratios, a binomial generalised linear model with a log link function was performed. Crude risk ratios (CRRs) were obtained using univariable regression analysis, while adjusted risk ratios (ARRs) were derived from multivariable regression analysis, adjusting for sex, age, hypertension, smoking, sleep, moderate exercise, and musculoskeletal condition.

Predictor	Crude RR		Adjusted RR	
	RR (95% CI)	*p*-Value	RR (95% CI)	*p*-Value
Female (vs. male)	1.43 (1.04–1.97)	0.03	1.49 (1.08–2.06)	0.015
Age (ref: >60)		0.153		0.062
<33	1.85 (0.83–4.11)	0.133	1.95 (0.89–4.24)	0.094
33–45	1.16 (0.65–2.06)	0.617	1.16 (0.66–2.04)	0.618
46–60	1.53 (0.86–2.72)	0.151	1.60 (0.91–2.82)	0.105
No hypertension (vs. Yes)	0.65 (0.46–0.93)	0.017	0.64 (0.46–0.89)	0.009
Musculoskeletal condition (No vs. Yes)	0.67 (0.50–0.91)	0.009	0.75 (0.56–1.01)	0.055
Moderate exercise (No vs. Yes)	1.17 (0.86–1.60)	0.313	1.19 (0.88–1.63)	0.26
Smoking (No vs. Yes)	0.78 (0.54–1.13)	0.197	0.66 (0.46–0.95)	0.027
<6 h Sleep	1.45 (1.08–1.94)	0.014	1.37 (1.02–1.84)	0.038

## Data Availability

The raw data supporting the conclusions of this article will be made available by the authors on request.

## Supplementary Materials

The following supporting information can be downloaded at: https://www.mdpi.com/article/10.3390/ijerph22101585/s1. Supplementary File S1: Questionnaire on Musculoskeletal Injuries of Hajj.
