# Supplementary material for: Prevalence and Risk Factors of Musculoskeletal Pain Among Kuwaiti Pilgrims During Hajj 2024
Source: Int J Environ Res Public Health. 2025 Oct 18;22(10):1585. doi: 10.3390/ijerph22101585 (PMC12562765; doi:10.3390/ijerph22101585)
Supplement: Supplementary file 1 [file ijerph-22-01585-s001.zip › ijerph-3867891-supplementary.pdf]

Serial #: ____________________________

Musculoskeletal Injuries of Hajj: A Cross-Sectional Study

Name:__________________________________ Date:_______________________

The purpose of this study is to report on the most common musculoskeletal injuries that may occur during Hajj. The information gathered from this study will serve to establish baseline data to help understand the most common injuries when performing Hajj activities and to help prevent such injuries in the future.

We will ask you to fill this questionnaire before the Hajj. We will then ask you to keep a record of any injuries during your Hajj pilgrimage. And finally, we will ask you to fill a final questionnaire after you return from the Hajj pilgrimage.

Your name is needed for us to cross reference your data before, during and after Hajj. Our code of ethics does require that your name is known only by the Team Leader for the project, Tahera Al-Eid, Department of Physical Therapy, Ministry of Health, Kuwait. No one else will have access to it. It is also important that you feel free to answer our questions as honestly as you can to make the data base meaningful and our findings useful in our making recommendations for your group. We believe that you may feel more comfortable if we ensure that your personal identity is not revealed. The data base will only identify you by code number.

Musculoskeletal Injuries of Hajj: A Cross-Sectional Study

<u>SECTION 1</u> Personal Data

Name		Marital Status	Married single divorced
Age		Number of children	
Gender		Highest level of education	High school Diploma Bsc Ms PhD , None
Height cm			
Weigh kg			
Occupation			
Phone			
Email			
Do you use	Smart Phone ☐ Yes ☐ No	Apple watch ☐ Yes ☐ No	Fitbit or smart band? ☐ Yes ☐ No

<u>SECTION 2</u> Health Status and Musculoskeletal Information

I am	underweight normal weight over weight I don't know
My heart rate is generally	low normal high I don't know
My blood pressure is	low normal high I don't know
My blood sugar is	low normal high I don't know
My heart rate is	low normal high I don't know
My general health is	Excellent Above average Average Below average Poor
Do you have a regular medical follow up?	☐ Yes ☐ No

Do you have any of the following conditions?

Condition	Yes	No
Heart disease		
Lung disease		
High blood pressure		
Stroke		
Obesity		
Diabetes		

Cancer		
Rheumatoid arthritis		
Osteoarthritis		
Osteoporosis		
Anemia		
Asthma		
Sickle cell anemia		
Multiple sclerosis		
Parkinson’s syndrome		
Irritable bowel syndrome		
Ulcers		
Women: Premenopausal		
Women: Menstrual irregularities		
Women: Menopausal		
Other		

What medications and/or vitamins and supplements do you currently take? ______________________________________

____

Please list any musculoskeletal conditions you have incurred such as muscle pulls, sprains, fractures, surgery, pain, or general discomfort:

Neck: ____

Upper Back: ____

Lower Back: ____

Hip / Pelvis: ____

Thigh / Knee: ____

Ankle: ____

Foot: ____

Other: ____

<u>SECTION 3</u> **Lifestyle behaviors**

A. Physical Activity and Exercise: Personal Behaviors

Rate your level of **regular** physical activity

1. I am moderately physically active for at least 3.5 hours during the week	☐ Yes ☐ No<br><br>If No, go to section B
2. My physical activity includes:<br><br>AEROBIC EXERCISE<br><br>Walking, running cycling or swimming at a **LOW** intensity (NO awareness of breathing or heart rate) at least for 20 to 30 minutes, 3 to 5 times a week	☐ Yes ☐ No<br><br>☐ Yes ☐ No

Walking, running cycling or swimming at a **MODERATE** intensity (aware of my breathing and heart rate) at least for 20 to 30 minutes, 3 to 5 times a week<br><br>STRENGTHENING (WEIGHTS OR BANDS)	
Weight, resistance band or machine work that is **LIGHT** (able to do more than 3 sets of 10 repetitions	☐ Yes ☐ No
Weight, resistance bands, or machine work that is **MODERATE/HEAVY** (can only do 3 sets of 10 repetitions)	☐ Yes ☐ No
OTHER ACTIVITIES<br>Other (specify____)<br>If yes, duration (_____________) and frequency (______________)	☐ Yes ☐ No

Type of shoes used during physical activity: _________________________________

Do you use a step counter on a daily basis? ☐ Yes ☐ No   If Yes, how many steps on average per day?___________

B. Smoking: Personal Behaviors

Do you smoke? ☐ Yes ☐ No

Present smoker If so, Cigarettes Sheesha Other (What?___________)

How many daily?____________For how long?________________

Past smoker but quit

Quit how many years ago?________ Cigarettes Sheesha Other

How many daily?____________For how long?________________

Lives with someone who smokes

For how long?_____________

Does this person smoke in your presence? ☐ Yes ☐ No

C. Sleep

Sleep:

I sleep:

Peacefully Poorly, causes of poor sleep:………………………………………………………

I sleep ……………….. hours a night on average.

POST-HAJJ

B PAIN and INJURY

Please list any musculoskeletal conditions you have incurred such as muscle pulls, sprains, fractures, surgery, pain, or general discomfort during Hajj:

Neck: ____

Upper Back: ____

Lower Back: ____

Hip / Pelvis: ____

Thigh / Knee: ____

Ankle: ____

Foot: ____

Other: ____

Did you take any medications for the pain or injury? If yes what is it? ______________________________

____

Did you use any assistance during Hajj? ______________________________

____

C. Sleep

Sleep:

I sleep:

Peacefully Poorly, causes of poor sleep:………………………………………………………

I sleep ……………….. hours a night on average.
